# Supplementary material for: Hot spot prediction in protein-protein interactions by an ensemble system
Source: BMC Syst Biol. 2018 Dec 31;12(Suppl 9):132. doi: 10.1186/s12918-018-0665-8 (PMC6311905; doi:10.1186/s12918-018-0665-8)
Supplement: Supplementary file 2 — All relevant MATLAB code. A simple Matlab implement of our predictor. (ZIP 19,742 kb) [file 12918_2018_665_MOESM2_ESM.zip › Additional file 2/Read ASA/ASA website/dbMPIKT/2NYYC.html]

 NetSurfP - Protein Surface Accessibility and Secondary Structure Predictions

  

|  |  |  |
| --- | --- | --- |
|  |  | NetSurfP - Protein Surface Accessibility and Secondary Structure Predictions  Technical University of Denmark |

  


---

```
# For publication of results, please cite:
# A generic method for assignment of reliability scores applied to solvent accessibility predictions.
# Bent Petersen, Thomas Nordahl Petersen, Pernille Andersen, Morten Nielsen and Claus Lundegaard
# BMC Structural Biology 2009, 9:51 doi:10.1186/1472-6807-9-51
#
# Column 1: Class assignment - B for buried or E for Exposed - Threshold: 25% exposure, but not based on RSA
# Column 2: Amino acid
# Column 3: Sequence name
# Column 4: Amino acid number
# Column 5: Relative Surface Accessibility - RSA
# Column 6: Absolute Surface Accessibility
# Column 7: Z-fit score for RSA prediction
# Column 8: Probability for Alpha-Helix
# Column 9: Probability for Beta-strand
# Column 10: Probability for Coil
E E  2NYYC                  1    0.800 139.743   0.717   0.003   0.003   0.994
B I  2NYYC                  2    0.255  47.119  -0.914   0.004   0.616   0.381
E V  2NYYC                  3    0.442  67.966   1.356   0.001   0.900   0.099
B L  2NYYC                  4    0.137  25.011   0.358   0.001   0.900   0.099
E T  2NYYC                  5    0.435  60.362   0.476   0.002   0.816   0.182
B Q  2NYYC                  6    0.142  25.415   0.408   0.004   0.514   0.481
E S  2NYYC                  7    0.371  43.481   0.281   0.004   0.138   0.858
E P  2NYYC                  8    0.502  71.262  -0.953   0.018   0.047   0.935
E A  2NYYC                  9    0.567  62.450  -1.227   0.018   0.088   0.893
E T  2NYYC                 10    0.401  55.591  -1.449   0.021   0.451   0.528
B L  2NYYC                 11    0.258  47.295  -0.374   0.021   0.756   0.223
E S  2NYYC                 12    0.490  57.405  -0.477   0.018   0.846   0.136
B L  2NYYC                 13    0.194  35.485   0.307   0.021   0.756   0.223
E S  2NYYC                 14    0.426  49.904   0.572   0.004   0.420   0.576
E P  2NYYC                 15    0.513  72.837   0.118   0.004   0.085   0.910
E G  2NYYC                 16    0.660  51.958  -1.005   0.005   0.015   0.979
E E  2NYYC                 17    0.500  87.315   0.470   0.004   0.138   0.858
E R  2NYYC                 18    0.543 124.439   0.914   0.004   0.616   0.381
B A  2NYYC                 19    0.076   8.386   0.118   0.001   0.900   0.099
E T  2NYYC                 20    0.430  59.683   1.499   0.001   0.959   0.040
B I  2NYYC                 21    0.056  10.304   0.346   0.001   0.959   0.040
E S  2NYYC                 22    0.322  37.727   0.807   0.001   0.900   0.099
B C  2NYYC                 23    0.025   3.496   0.427   0.001   0.900   0.099
E R  2NYYC                 24    0.373  85.371   1.267   0.001   0.900   0.099
B A  2NYYC                 25    0.168  18.514  -1.135   0.001   0.900   0.099
B S  2NYYC                 26    0.297  34.808  -0.808   0.003   0.718   0.279
E E  2NYYC                 27    0.476  83.087  -2.085   0.022   0.359   0.619
E S  2NYYC                 28    0.387  45.380  -2.279   0.020   0.205   0.775
E V  2NYYC                 29    0.394  60.573  -1.513   0.022   0.359   0.619
E D  2NYYC                 30    0.463  66.747  -1.788   0.021   0.279   0.699
E S  2NYYC                 31    0.386  45.263  -2.456   0.019   0.141   0.840
E Y  2NYYC                 32    0.429  91.613  -1.930   0.004   0.085   0.910
E G  2NYYC                 33    0.494  38.917  -1.826   0.004   0.085   0.910
E H  2NYYC                 34    0.358  65.193  -0.898   0.004   0.197   0.799
B S  2NYYC                 35    0.240  28.163  -0.382   0.004   0.420   0.576
B F  2NYYC                 36    0.125  25.108   0.865   0.003   0.718   0.279
B M  2NYYC                 37    0.025   5.063   0.679   0.001   0.900   0.099
B Q  2NYYC                 38    0.088  15.645   0.346   0.011   0.918   0.071
B W  2NYYC                 39    0.030   7.287   0.310   0.011   0.918   0.071
B Y  2NYYC                 40    0.061  13.143   0.576   0.011   0.918   0.071
B Q  2NYYC                 41    0.150  26.790   0.601   0.002   0.816   0.182
B Q  2NYYC                 42    0.150  26.754   0.404   0.004   0.616   0.381
E K  2NYYC                 43    0.424  87.176   1.693   0.004   0.085   0.910
E P  2NYYC                 44    0.533  75.562  -0.330   0.005   0.015   0.979
E G  2NYYC                 45    0.772  60.717  -2.147   0.005   0.015   0.979
E Q  2NYYC                 46    0.507  90.550  -0.029   0.018   0.047   0.935
E A  2NYYC                 47    0.324  35.650  -0.442   0.004   0.085   0.910
B P  2NYYC                 48    0.113  16.035  -0.136   0.005   0.262   0.733
E R  2NYYC                 49    0.268  61.303   0.424   0.003   0.718   0.279
B L  2NYYC                 50    0.085  15.545   0.347   0.001   0.900   0.099
B L  2NYYC                 51    0.036   6.592  -0.113   0.001   0.959   0.040
B I  2NYYC                 52    0.023   4.163   0.230   0.001   0.959   0.040
B Y  2NYYC                 53    0.075  16.070   0.028   0.001   0.959   0.040
B R  2NYYC                 54    0.103  23.679  -0.772   0.001   0.900   0.099
B A  2NYYC                 55    0.119  13.103  -0.854   0.004   0.514   0.481
B S  2NYYC                 56    0.204  23.932  -1.005   0.020   0.205   0.775
E N  2NYYC                 57    0.511  74.840  -1.381   0.018   0.088   0.893
E L  2NYYC                 58    0.393  71.977  -0.924   0.018   0.047   0.935
E E  2NYYC                 59    0.618 108.017  -1.699   0.018   0.047   0.935
E P  2NYYC                 60    0.618  87.708  -1.580   0.018   0.088   0.893
E G  2NYYC                 61    0.565  44.434  -1.013   0.018   0.088   0.893
E I  2NYYC                 62    0.313  57.905  -0.332   0.005   0.045   0.951
E P  2NYYC                 63    0.619  87.850  -0.713   0.005   0.045   0.951
E A  2NYYC                 64    0.447  49.226  -1.804   0.004   0.138   0.858
E R  2NYYC                 65    0.329  75.364  -0.603   0.004   0.514   0.481
B F  2NYYC                 66    0.112  22.458  -1.242   0.003   0.718   0.279
B S  2NYYC                 67    0.267  31.316  -0.938   0.003   0.718   0.279
E G  2NYYC                 68    0.335  26.396  -2.085   0.003   0.718   0.279
E S  2NYYC                 69    0.403  47.208  -1.415   0.004   0.616   0.381
E G  2NYYC                 70    0.450  35.423  -1.023   0.004   0.514   0.481
E S  2NYYC                 71    0.483  56.596  -1.671   0.005   0.262   0.733
E G  2NYYC                 72    0.574  45.213  -1.274   0.004   0.138   0.858
E T  2NYYC                 73    0.386  53.566  -0.995   0.004   0.420   0.576
E D  2NYYC                 74    0.398  57.323   0.415   0.002   0.816   0.182
B F  2NYYC                 75    0.076  15.253  -0.674   0.001   0.900   0.099
B T  2NYYC                 76    0.258  35.771   0.268   0.001   0.959   0.040
B L  2NYYC                 77    0.035   6.409   0.203   0.001   0.959   0.040
B T  2NYYC                 78    0.248  34.453   0.359   0.001   0.959   0.040
B I  2NYYC                 79    0.068  12.617   0.004   0.001   0.900   0.099
E S  2NYYC                 80    0.360  42.157   0.308   0.002   0.816   0.182
E S  2NYYC                 81    0.348  40.821  -0.997   0.004   0.514   0.481
E L  2NYYC                 82    0.433  79.264  -0.503   0.005   0.262   0.733
E E  2NYYC                 83    0.524  91.508   0.404   0.004   0.197   0.799
E P  2NYYC                 84    0.369  52.347  -0.311   0.005   0.262   0.733
E E  2NYYC                 85    0.602 105.222  -1.125   0.005   0.262   0.733
E D  2NYYC                 86    0.604  86.993  -0.356   0.005   0.336   0.660
E F  2NYYC                 87    0.401  80.400  -0.735   0.004   0.514   0.481
B A  2NYYC                 88    0.255  28.101  -0.526   0.003   0.718   0.279
E V  2NYYC                 89    0.336  51.689   0.289   0.001   0.900   0.099
B Y  2NYYC                 90    0.067  14.382   1.082   0.001   0.900   0.099
B Y  2NYYC                 91    0.131  27.931   1.194   0.001   0.900   0.099
B C  2NYYC                 92    0.036   4.998  -0.313   0.001   0.900   0.099
B Q  2NYYC                 93    0.126  22.539  -0.261   0.002   0.816   0.182
B Q  2NYYC                 94    0.101  18.003  -0.456   0.023   0.655   0.322
B G  2NYYC                 95    0.226  17.786  -0.694   0.021   0.451   0.528
B N  2NYYC                 96    0.299  43.818  -1.041   0.021   0.279   0.699
E E  2NYYC                 97    0.372  64.901  -1.573   0.056   0.142   0.802
E V  2NYYC                 98    0.372  57.130  -1.966   0.056   0.142   0.802
E P  2NYYC                 99    0.403  57.143  -1.354   0.052   0.084   0.864
E F  2NYYC                100    0.373  74.821  -1.287   0.052   0.084   0.864
E T  2NYYC                101    0.339  46.992  -1.083   0.052   0.084   0.864
B F  2NYYC                102    0.284  57.079  -1.612   0.056   0.142   0.802
E G  2NYYC                103    0.365  28.718  -1.623   0.064   0.216   0.721
E Q  2NYYC                104    0.340  60.724  -1.111   0.066   0.296   0.638
E G  2NYYC                105    0.334  26.246  -0.831   0.125   0.227   0.648
E T  2NYYC                106    0.312  43.205  -1.017   0.118   0.150   0.732
E K  2NYYC                107    0.353  72.694  -1.143   0.118   0.150   0.732
E V  2NYYC                108    0.323  49.630  -1.319   0.118   0.150   0.732
E E  2NYYC                109    0.346  60.376  -0.980   0.118   0.150   0.732
E I  2NYYC                110    0.367  67.932  -1.537   0.125   0.227   0.648
E K  2NYYC                111    0.374  76.911  -0.989   0.125   0.227   0.648
E R  2NYYC                112    0.359  82.303  -1.607   0.066   0.296   0.638
E T  2NYYC                113    0.387  53.746  -1.067   0.066   0.296   0.638
B V  2NYYC                114    0.305  46.894  -1.711   0.066   0.296   0.638
E A  2NYYC                115    0.416  45.843  -1.468   0.066   0.296   0.638
E A  2NYYC                116    0.390  42.956  -1.559   0.069   0.386   0.545
E P  2NYYC                117    0.435  61.684  -1.721   0.022   0.359   0.619
E S  2NYYC                118    0.381  44.618  -1.036   0.022   0.359   0.619
B V  2NYYC                119    0.306  47.063  -1.908   0.022   0.359   0.619
E F  2NYYC                120    0.404  81.063  -1.598   0.021   0.279   0.699
E I  2NYYC                121    0.436  80.678  -2.145   0.019   0.141   0.840
B F  2NYYC                122    0.275  55.253  -2.035   0.004   0.085   0.910
E P  2NYYC                123    0.362  51.368  -1.784   0.005   0.015   0.979
B P  2NYYC                124    0.272  38.654  -2.301   0.005   0.015   0.979
B S  2NYYC                125    0.278  32.593  -1.959   0.018   0.019   0.964
E D  2NYYC                126    0.430  62.006  -1.802   0.018   0.047   0.935
E E  2NYYC                127    0.481  83.943  -2.138   0.018   0.047   0.935
E Q  2NYYC                128    0.393  70.261  -2.153   0.018   0.047   0.935
B L  2NYYC                129    0.258  47.185  -1.246   0.018   0.047   0.935
E K  2NYYC                130    0.541 111.222  -0.882   0.018   0.047   0.935
E S  2NYYC                131    0.567  66.464  -1.316   0.018   0.047   0.935
E G  2NYYC                132    0.494  38.909  -1.563   0.018   0.088   0.893
E T  2NYYC                133    0.442  61.236  -0.733   0.005   0.336   0.660
B A  2NYYC                134    0.169  18.668  -0.692   0.003   0.718   0.279
B S  2NYYC                135    0.187  21.881  -0.174   0.001   0.900   0.099
B V  2NYYC                136    0.055   8.500   0.479   0.001   0.900   0.099
B V  2NYYC                137    0.050   7.608  -0.099   0.001   0.959   0.040
B C  2NYYC                138    0.017   2.443   0.950   0.001   0.959   0.040
B L  2NYYC                139    0.123  22.448   0.306   0.001   0.959   0.040
B L  2NYYC                140    0.030   5.493  -0.567   0.002   0.816   0.182
B N  2NYYC                141    0.278  40.626   0.041   0.004   0.514   0.481
E N  2NYYC                142    0.555  81.208  -1.142   0.004   0.197   0.799
B F  2NYYC                143    0.243  48.790  -1.448   0.004   0.138   0.858
E Y  2NYYC                144    0.278  59.494  -0.395   0.004   0.138   0.858
E P  2NYYC                145    0.323  45.820  -1.841   0.004   0.085   0.910
E R  2NYYC                146    0.594 135.957  -1.361   0.004   0.138   0.858
E E  2NYYC                147    0.418  72.990  -1.236   0.005   0.336   0.660
B A  2NYYC                148    0.104  11.439   0.046   0.002   0.816   0.182
B K  2NYYC                149    0.236  48.463   0.488   0.001   0.900   0.099
B V  2NYYC                150    0.039   6.010  -0.123   0.001   0.959   0.040
B Q  2NYYC                151    0.164  29.237   0.624   0.011   0.918   0.071
B W  2NYYC                152    0.044  10.558   0.226   0.011   0.918   0.071
E K  2NYYC                153    0.267  54.901   0.808   0.001   0.900   0.099
E V  2NYYC                154    0.321  49.261   0.513   0.003   0.718   0.279
E D  2NYYC                155    0.634  91.417   0.082   0.020   0.205   0.775
E N  2NYYC                156    0.614  89.890  -1.331   0.005   0.015   0.979
E A  2NYYC                157    0.665  73.250  -0.825   0.005   0.045   0.951
E L  2NYYC                158    0.684 125.259  -2.376   0.004   0.138   0.858
E Q  2NYYC                159    0.452  80.745  -0.813   0.004   0.085   0.910
E S  2NYYC                160    0.448  52.506  -1.412   0.005   0.045   0.951
E G  2NYYC                161    0.586  46.102  -2.074   0.005   0.045   0.951
E N  2NYYC                162    0.529  77.431  -1.768   0.004   0.197   0.799
E S  2NYYC                163    0.381  44.688  -1.256   0.004   0.514   0.481
E Q  2NYYC                164    0.319  56.920  -0.342   0.023   0.655   0.322
E E  2NYYC                165    0.519  90.722  -1.515   0.022   0.552   0.426
E S  2NYYC                166    0.412  48.275  -2.032   0.021   0.279   0.699
E V  2NYYC                167    0.412  63.371  -1.517   0.020   0.205   0.775
E T  2NYYC                168    0.362  50.209  -0.418   0.021   0.279   0.699
E E  2NYYC                169    0.342  59.712  -1.557   0.021   0.279   0.699
E Q  2NYYC                170    0.531  94.854  -1.514   0.021   0.279   0.699
E D  2NYYC                171    0.497  71.560  -1.494   0.021   0.279   0.699
E S  2NYYC                172    0.384  44.958  -1.943   0.020   0.205   0.775
E K  2NYYC                173    0.607 124.798  -1.454   0.005   0.045   0.951
E D  2NYYC                174    0.758 109.285  -2.533   0.005   0.015   0.979
E S  2NYYC                175    0.435  51.005  -1.899   0.005   0.045   0.951
E T  2NYYC                176    0.345  47.810  -0.426   0.005   0.336   0.660
B Y  2NYYC                177    0.122  26.114  -1.274   0.004   0.514   0.481
B S  2NYYC                178    0.116  13.595  -2.137   0.003   0.718   0.279
B L  2NYYC                179    0.099  18.200  -1.201   0.002   0.816   0.182
B S  2NYYC                180    0.180  21.073  -0.750   0.001   0.900   0.099
B S  2NYYC                181    0.044   5.122  -1.454   0.001   0.959   0.040
B T  2NYYC                182    0.177  24.605  -0.066   0.001   0.959   0.040
B L  2NYYC                183    0.043   7.855  -0.109   0.001   0.900   0.099
E T  2NYYC                184    0.311  43.080   0.843   0.002   0.816   0.182
B L  2NYYC                185    0.065  11.938   0.291   0.003   0.718   0.279
E S  2NYYC                186    0.353  41.407   1.191   0.021   0.451   0.528
E K  2NYYC                187    0.445  91.536  -0.745   0.021   0.279   0.699
E A  2NYYC                188    0.534  58.825  -0.923   0.021   0.279   0.699
E D  2NYYC                189    0.535  77.065   0.619   0.020   0.205   0.775
B Y  2NYYC                190    0.169  36.158  -0.849   0.019   0.141   0.840
E E  2NYYC                191    0.662 115.599   0.510   0.005   0.045   0.951
E K  2NYYC                192    0.654 134.569   0.684   0.005   0.045   0.951
E H  2NYYC                193    0.616 111.959  -0.392   0.005   0.045   0.951
E K  2NYYC                194    0.540 111.037   0.258   0.005   0.336   0.660
E V  2NYYC                195    0.437  67.167   1.698   0.002   0.816   0.182
B Y  2NYYC                196    0.060  12.907   1.087   0.001   0.959   0.040
E A  2NYYC                197    0.312  34.371   1.796   0.001   0.959   0.040
B C  2NYYC                198    0.024   3.327   0.768   0.001   0.959   0.040
B E  2NYYC                199    0.258  45.125   1.297   0.001   0.900   0.099
B V  2NYYC                200    0.093  14.263   0.399   0.001   0.900   0.099
E T  2NYYC                201    0.447  61.999   0.499   0.001   0.959   0.040
B H  2NYYC                202    0.142  25.921   0.531   0.002   0.816   0.182
E Q  2NYYC                203    0.645 115.179   0.076   0.005   0.262   0.733
E G  2NYYC                204    0.621  48.912  -1.709   0.005   0.045   0.951
E L  2NYYC                205    0.394  72.141  -0.862   0.005   0.045   0.951
E S  2NYYC                206    0.679  79.555   0.144   0.005   0.045   0.951
E S  2NYYC                207    0.553  64.753   0.222   0.018   0.088   0.893
E P  2NYYC                208    0.446  63.259   0.172   0.021   0.279   0.699
B V  2NYYC                209    0.177  27.174  -0.404   0.003   0.718   0.279
E T  2NYYC                210    0.548  75.952   0.452   0.001   0.900   0.099
E K  2NYYC                211    0.247  50.746   1.198   0.001   0.900   0.099
E S  2NYYC                212    0.462  54.158   0.145   0.001   0.900   0.099
B F  2NYYC                213    0.109  21.816   0.733   0.001   0.900   0.099
E N  2NYYC                214    0.505  73.917   1.597   0.004   0.616   0.381
E R  2NYYC                215    0.448 102.615   0.339   0.020   0.205   0.775
E G  2NYYC                216    0.650  51.186  -1.222   0.019   0.141   0.840
E E  2NYYC                217    0.654 114.184   1.476   0.018   0.088   0.893
E C  2NYYC                218    0.480  67.392  -1.320   0.003   0.003   0.994

Explain the output.  Go back.
```

---
